# Exercise Capacity and the Force Frequency Relationship in Multi‐Point Versus Single‐Point Pacing: A Randomized Trial

**DOI:** 10.1111/pace.70164

**Published:** 2026-02-20

**Authors:** Nawaz Z. Safdar, Ria M. Gadani, Charlotte A. Cole, Judith E. Lowry, Stephe Kamalathasan, Sushma Datla, Oliver I. Brown, Nithusa Rahunathan, Maria F. Paton, Mark T. Kearney, Sam Straw, Klaus K. Witte, John Gierula

**Affiliations:** ^1^ The Leeds Teaching Hospitals NHS Trust Leeds UK; ^2^ Pennsylvania Hospital, The University of Pennsylvania Health System Philadelphia USA; ^3^ The Northern Lincolnshire and Goole NHS Trust Scunthorpe UK; ^4^ Leeds Institute for Cardiovascular and Metabolic Medicine University of Leeds Leeds UK

**Keywords:** cardiac resynchronization therapy, contractility, heart failure, multipoint pacing, pacemakers

## Abstract

**Background:**

Quadripolar left ventricular (LV) epicardial leads capable of multipoint pacing (MPP) may have an advantage over conventional bipolar leads for delivering cardiac resynchronization therapy (CRT) by stimulating the lateral LV wall from two distinct locations simultaneously.

**Aim:**

We aimed to determine the acute and longer‐term effects of MPP compared with single‐point pacing (SPP) on LV contractility and exercise capacity in individuals with heart failure with reduced ejection fraction receiving CRT.

**Methods:**

Participants were enrolled into a randomized crossover study with echocardiographic assessment of the comparative effects of acute MPP and SPP on LV contractility and cardiopulmonary exercise testing at 6‐weeks and 6‐months following device implantation. Participants were then randomized in a parallel‐group study to either MPP or SPP for further 6‐months.

**Results:**

Twenty‐three participants (mean age 73 years [95% confidence interval: 69, 78], 91% male, 91% New York Heart Association [NYHA] class II, LV ejection fraction 31.3% [27.4, 35.1]) were included. At resting heart rates, LV contractility was significantly higher with MPP compared to SPP (2.29 mmHg/mL/m^2^ [1.74, 2.84] vs. 2.03 [1.58, 2.47]; *p* = 0.019). However, it was not different between MPP and SPP at higher heart rates or at 6‐months, and there were no differences in exercise performance between MPP and SPP at any point including following 6 months of chronic treatment.

**Conclusion:**

Although CRT with MPP resulted in improved LV contractility at resting heart rates acutely post implantation, it did not translate into consistent mechanistic or patient‐orientated benefits in the short or longer‐term.

**Abbreviations**
CCI: cardiac contractility index
CI: confidence interval
CRT: cardiac resynchronization therapy
FFR: force‐frequency relationship
HFrEF: heart failure with reduced ejection fraction
LV: left ventricular
LVEF: left ventricular ejection fraction
MPP: multipoint pacing
NYHA: New York Heart Association
OUES: oxygen uptake efficiency slope
RER: respiratory exchange ratio
SPP: single‐point pacing
V̇CO_2_
: carbon dioxide output
V̇O_2_
: oxygen consumption

## 1 Introduction

### 1.1 Background

Cardiac resynchronization therapy (CRT) improves symptoms, reduces hospitalization, and extends longevity for individuals who have heart failure, persistent left ventricular (LV) systolic dysfunction, and left bundle branch block [1, 2, 3, 4, 5]. Traditional LV leads were capable of delivering only bipolar pacing, such that a proposed contributor of poor response in some patients was the anatomical location of the lead cathode [6, 7]. Consequently, quadripolar leads with multiple options for cathode location have been developed. These leads can stimulate the lateral LV epicardium at two cathodal sites simultaneously, known as multipoint pacing (MPP).

It has been observed that MPP is associated with acute improvements in LV contractility and hemodynamics compared with single‐point pacing (SPP) [8, 9, 10]. However, studies which have explored these hemodynamic effects of MPP did so whilst participants were at rest, and at a single (resting) heart rate (HR). In addition, the effects on patient‐orientated outcomes have been equivocal [11]. In the context of decreased battery longevity that may lead to earlier generator replacement [12, 13], the evidence supporting widespread clinical adoption of MPP remains unclear. If MPP were to translate into improvements in patient‐orientated outcomes in conjunction with improved coordination and cardiac contractility of the left ventricle, one would expect this to also be the case at higher HRs.

### 1.2 Aims

We therefore aimed to describe the acute effects of activating MPP on the force‐frequency relationship (FFR) and exercise capacity shortly (6 weeks) and six months following CRT implantation for heart failure with intraventricular conduction delay. Next, we aimed to explore the effects of longer term (6 months) MPP delivery on exercise capacity.

## 2 Methods

### 2.1 Study Design

The Optimizing Pacemaker Therapy with multipoint Pacing (OPT‐MPP) study assessed the acute and longer‐term effects of MPP on the FFR and exercise capacity in patients with heart failure with reduced ejection fraction (HFrEF) implanted with a *de novo* CRT device for heart failure in two blinded crossover phases and one longer term double‐blind parallel group phase.

### 2.2 Participants

Consecutive patients undergoing the implantation of CRT devices with quadripolar LV leads (Table S1), with or without a defibrillator at a single center between July 2017 and February 2019 were approached to participate. Patients were eligible for inclusion if they had symptomatic heart failure, LV ejection fraction (LVEF) <35%, and a conduction abnormality with a *de novo* CRT device with a quadripolar LV lead. Exclusion criteria were the inability to perform a cardiopulmonary exercise test due to respiratory or neuromuscular limitation including significant arthritis. We also excluded patients with atrial fibrillation with a poorly controlled ventricular response, symptoms of angina pectoris that limited exercise tolerance, unstable symptoms of heart failure or changes to medical therapy within the preceding three months, poor echocardiography images precluding the measurement of LV volumes, and concurrent use of calcium channel blockers.

### 2.3 Study Phases

**FIGURE 1:**
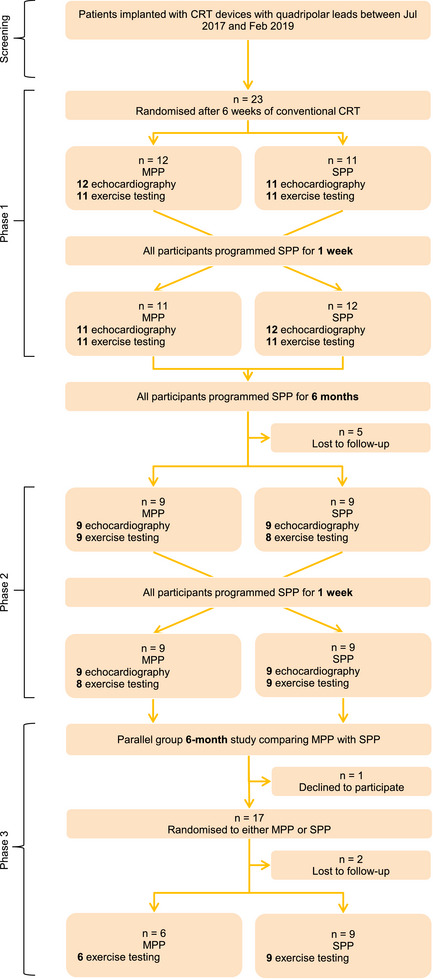
OPT‐MPP flow diagram. CRT, cardiac resynchronization therapy; MPP, multipoint pacing; SPP, single‐point pacing. [Colour figure can be viewed at wileyonlinelibrary.com]

The study consisted of three distinct phases, which are summarized in Figure 1. In phase 1, the acute effects of MPP activation were assessed in a double‐blind, crossover design shortly (6 weeks) following device implantation. In phase 2, this design was repeated at 6 months after implantation to assess the effects of MPP following cardiac remodeling with a CRT device. In phase 3, we assessed the longer‐term (6 month) effects of MPP on patient orientated outcomes in a double‐blind, controlled, parallel group design starting at 6 months following implantation after the completion of phase 2. At the initial screening visit, relevant comorbidities and prescribed medical therapy were recorded, and any changes during the study were also documented. All devices were interrogated at the start and end of each study visit to ensure safety and functionality. During study phases 1 and 2, all devices were programmed to SPP (usual care) between study visits. Programming choices beyond SPP or MPP, including the use or non‐use of AV delay optimization algorithms were at the discretion of the treating physician. However, the effects of these choices on outcomes are mitigated by the study's crossover design.

#### 2.3.1 Phase 1: Randomized, Double‐Blind, Crossover Study at 6‐Weeks

Approximately 6 weeks following device implantation, participants underwent two visits at the Leeds General Infirmary NIHR Clinical Research Facility one week apart. At each visit they were randomly allocated in a 1:1 pattern to have MPP activated or SPP programming maintained by an unblinded cardiac scientist. The participant and a second cardiac scientist (who performed echocardiography) remained blinded to group allocation. The FFR was assessed by echocardiography as previously described [14, 15] and the participant then performed a peak, symptom‐limited, treadmill‐based cardiopulmonary exercise test. At the end of each research visit, CRT devices were programmed back to their nominal bipolar CRT delivery (SPP). After one week, the study procedures were repeated with the device programmed to the opposite mode.

#### 2.3.2 Phase 2: Randomized, Double‐blind, Crossover Study at 6‐Months

After 6 months, participants attended for two further visits one week apart during which the procedures described for phase 1 were repeated. As in phase 1, between the visits, CRT delivery was maintained in bipolar (SPP) mode.

#### 2.3.3 Phase 3: Randomized Parallel‐group Study From 6‐Months After Implantation

At the end of the second visit completing phase 2, participants were invited to participate in a longer‐term, parallel‐group study where they were randomly allocated to either chronic MPP or maintained SPP programming for a further 6 months, following which they underwent a further assessment of exercise capacity.

### 2.4 Study Procedures

#### 2.4.1 Assessment of the Force‐Frequency Relationship

We determined the FFR noninvasively using transthoracic echocardiography as previously described [15]. Briefly, an accredited cardiac physiologist blinded to CRT programming acquired two and four‐chamber images using a Vivid E95 Ultrasound System (GE Healthcare, Chicago, IL) whilst the CRT device was programmed to increase the HR from the base rate at 15 beats/min increments to a maximum of 140 beats/min. Images were analyzed offline using the Echopac (GE Healthcare, Chicago, IL) digital imaging system. We measured LV end‐diastolic and end‐systolic volumes using the biplane (modified Simpson's) method of discs by tracing the endocardial border, excluding the papillary muscles, on the frame captured at the start of the R‐wave, and the frame with the smallest LV cavity, respectively. The average of measurements taken over three cardiac cycles was used in the final analysis, and volumes were indexed for body surface area (BSA) using the Mostellar equation.

At each HR, LV contractility was determined as the ratio between LV end‐systolic pressure and the LV end‐systolic volume, divided by BSA to achieve a standardized cardiac contractility index (CCI). Systolic blood pressure was used as a surrogate for LV end‐systolic pressure, as previously described [16, 17, 18]. Blood pressure was recorded using a sphygmomanometer and a standard stethoscope at rest and at each incremental HR. We defined the “critical heart rate” as the HR at which LV contractility peaked.

#### 2.4.2 Cardiopulmonary Exercise Testing

Participants underwent cardiopulmonary exercise testing using a treadmill ramp protocol, as previously described, with 3‐min incremental stages [19]. Metabolic gas exchange analysis was performed using collected exhaled air during each test (Ultimo CardO2, Medical Graphics, St. Paul, Minnesota). HR (beats/min), oxygen consumption (V̇O_2_), and carbon dioxide output (V̇CO_2_) were recorded every 15s. The anaerobic threshold was calculated using the V‐slope method [20]. Equipment was recalibrated prior to each test. All test subjects were encouraged to exercise to exhaustion, and no further instructions were given. We arranged the laboratory with a curtain between the ECG monitor and the patient to ensure double‐blinding (of both the participant and the supervising cardiac scientist), whilst providing ECG monitoring by the unblinded cardiac scientist as previously described [14, 15].

### 2.5 Statistical Analyses

Continuous data are presented as means and their 95% confidence intervals (CIs), whereas discrete variables are presented as numbers and percentages. Normality of distribution of the continuous data was assessed visually by distribution plots and confirmed using skewness testing. Statistical comparisons for normally distributed variables were by paired and unpaired Student's *t*‐test as appropriate. Analysis of covariance (adjusted by LV ejection fraction, end systolic volume, and end diastolic volume) was used to assess intergroup differences in change in exercise parameters between phases 2 and 3. Statistical significance was defined as a *p*‐value of < 0.05 and all tests were two‐sided. No imputation was made for missing data. Statistical analyses were done using Stata (StataCorp. 2019. Stata Statistical Software: Release 16. College Station, TX: StataCorp LLC.), with GraphPad Prism (version 8.0.1 for Windows, GraphPad Software, Boston, Massachusetts USA, www.graphpad.com) used for illustrations.

### 2.6 Ethical Considerations

The study was sponsored by the University of Leeds and received favorable opinion from the Health Research Authority following review by the Southwest—Central Bristol Research Ethics Committee (external reference: 17/SW/0200, date of opinion: 14/09/2017). All participants provided informed written consent prior to any research activity taking place. The study was conducted in accordance with the principals of the Declaration of Helsinki.

## 3 Results

A total of 23 patients who had CRT devices with quadripolar LV leads agreed to participate. Baseline clinical and echocardiographic characteristics of the entire cohort are summarized in Table 1.

**TABLE 1:** The baseline clinical and echocardiographic characteristics of the 23 participants at the start of the study period.

Characteristic	
Age (mean, 95CI), years	73 (69, 78)
Male sex (*n*, %)	21 (91.3)
Height (mean, 95CI), m	1.74 (1.71, 1.77)
Weight (mean, 95CI), kg	86.6 (80.1, 93.1)
BSA (mean, 95CI), m^2^	2.04 (1.96, 2.13)
NYHA class at baseline (*n*, %)	
Class I	1 (4.3)
Class II	21 (91.3)
Class III	3 (4.3)
Class IV	0
Past medical history (*n*, %)	
Ischaemic heart disease	13 (56.5)
Persistent atrial fibrillation	7 (30.4)
Hypertension	11 (47.8)
Diabetes mellitus	9 (39.1)
Device type (*n*, %)	
CRT‐D	16 (69.6)
CRT‐P	7 (30.4)

Conduction abnormality (*n*, %)	
LBBB	20 (87.0)
RBBB	2 (8.7)
IBBB	1 (4.3)
Medical treatment (*n*, %)	
Beta‐blockers	23 (100)
ACE inhibitors/ARBs	21 (91.3)
MRA	16 (69.6)
Diuretics	11 (47.8)
Echocardiographic findings (mean, 95CI)	
LVEF, %	31.3 (27.4, 35.1)
LVEDV, mL	202.7 (166.4, 239.0)
LVESV, mL	142.0 (111.6, 172.3)

Abbreviations: MPP, multipoint pacing; SPP, single‐point pacing; 95CI, 95% confidence interval; BSA, body‐surface area; NYHA, New York Heart Association; CRT‐D, cardiac resynchronization therapy defibrillator; CRT‐P, cardiac resynchronization therapy pacemaker; ACE, angiotensin‐converting enzyme; ARB, angiotensin II receptor blocker; MRA, mineralocorticoid receptor antagonist; LVEF, left ventricular ejection fraction; LVEDV, left ventricular end diastolic volume; LVESV, left ventricular end systolic volume.

### 3.1 Phase 1

#### 3.1.1 Force‐Frequency Relationship

**FIGURE 2:**
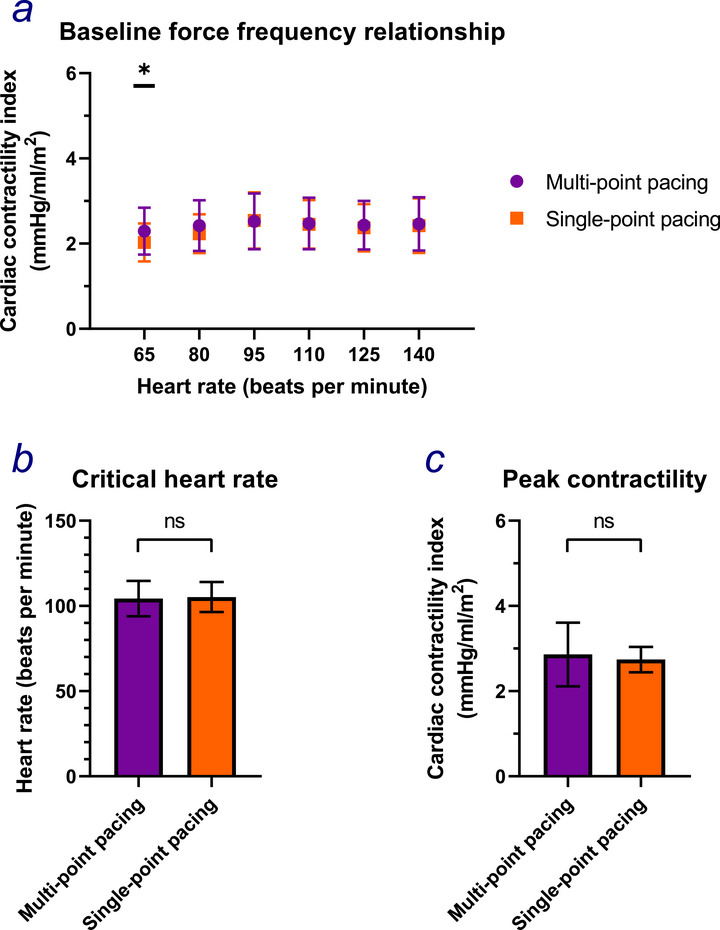
The differences in (a) the Cardiac Contractility Index at heart rate increments of 15 beats/min starting at 65 up until 140; (b) the critical heart rate; (c) the peak contractility between multipoint pacing (purple) and single‐point pacing (orange) during phase 1 of OPT‐MPP. Paired Student's *t*‐test. *, *p* = 0.019; ns, *p* > 0.05. [Colour figure can be viewed at wileyonlinelibrary.com]

At six weeks, LV contractility was higher during MPP stimulation than during SPP stimulation at rest, specifically 65 beats/min (mean CCI 2.29 mmHg/mL/m^2^ [CI 1.74, 2.84] vs. 2.03 [1.58, 2.47]; *p* = 0.02) (Table S2, Figure 2a). However, there was no difference in contractility between the two modes at higher HRs. Neither the HR at which peak contractility occurred (MPP: mean 104 bpm [CI 94, 115] vs. SPP: 105 bpm [96, 114]; *p* = 0.90; Figure 2b) or LV contractility at this HR (MPP: mean 2.86 mmHg/mL/m^2^ [CI 2.11, 3.61] vs. SPP: 2.74 [2.12, 3.35]; *p* = 0.33; Figure 2c) were different between the programming modes.

#### 3.1.2 Cardiopulmonary Exercise Testing

**FIGURE 3:**
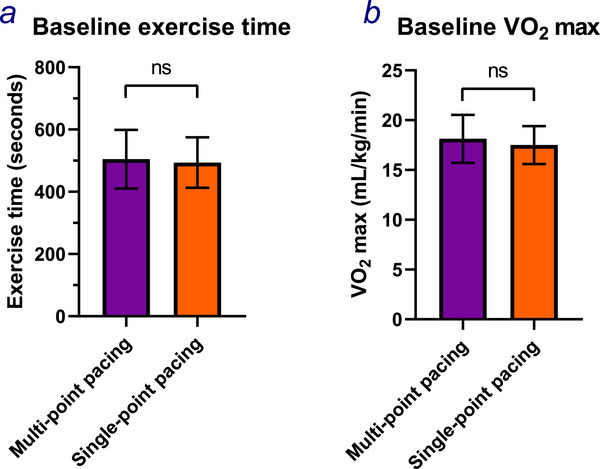
The differences in (a) the exercise time in seconds; (b) the maximal oxygen consumption between multipoint pacing (purple) and single‐point pacing (orange) during phase 1 of OPT‐MPP. Paired Student's *t*‐test. ns, *p* > 0.05. [Colour figure can be viewed at wileyonlinelibrary.com]

Exercise testing was performed for 22 participants at the first phase (six weeks post implantation). There were no differences in resting or peak HR, resting systolic or diastolic blood pressure, exercise time (Figure 3a), V̇O_2_ max (Figure 3b), oxygen uptake efficiency slope (OUES), anaerobic threshold, V̇E/V̇CO_2_ slope or V̇E/V̇CO_2_ slope to anaerobic threshold, or peak respiratory exchange ratio (RER) between the tests done with MPP active versus those done with SPP (Table 2).

**TABLE 2:** The differences in cardiopulmonary exercise testing between multipoint and single‐point pacing during phase 1 of OPT‐MPP.

CPET, mean (95CI)	MPP	SPP	*p*‐value
Resting HR, bpm	67.5 (62.8, 72.2)	63.8 (60.3, 67.2)	0.19
Peak HR, bpm	108.6 (98.6, 118.6)	106.4 (99.1, 113.7)	0.72
Resting systolic BP, mmHg	125.6 (116.1, 135.2)	124.6 (112.1, 137.2)	0.89
Resting diastolic BP, mmHg	69.1 (65.4, 72.7)	69.2 (62.6, 75.9)	0.96
Exercise time, seconds	504.8 (410.7, 599.0)	493.9 (412.9, 574.9)	0.86
VO_2_ max, mL/kg/min	18.1 (15.7, 20.5)	17.5 (15.6, 19.4)	0.67
OUES	1885.5 (1625.4, 2145.5)	1872.9 (1605.6, 2140.1)	0.94
Anaerobic threshold	14.5 (12.6, 16.5)	13.7 (12.1, 15.4)	0.52
Ve/VCO_2_ slope	33.2 (29.8, 36.6)	32.1 (28.8, 35.5)	0.64
Ve/VCO_2_ slope to AT	30.6 (27.1, 34.1)	29.1 (25.8, 32.4)	0.52
Peak RER	1.04 (0.98, 1.09)	1.08 (1.02, 1.14)	0.29

*Note*: Paired Student's *t*‐test for statistical comparisons.

Abbreviations: CPET, cardiopulmonary exercise test; 95CI, 95% confidence interval; MPP, multipoint pacing; SPP, single‐point pacing; HR, heart rate; BP, blood pressure; OUES, oxygen uptake efficiency slope; AT, anaerobic threshold; RER, respiratory exchange ratio.

### 3.2 Phase 2

#### 3.2.1 Force‐Frequency Relationship

**FIGURE 4:**
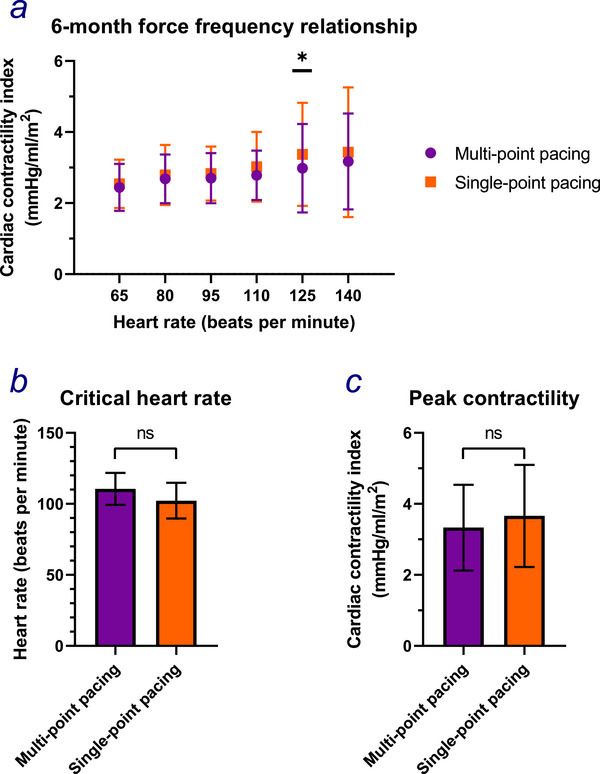
The differences in (a) the Cardiac Contractility Index at heart rate increments of 15 beats/min starting at 65 up until 140; (b) the critical heart rate; (c) the peak contractility between multipoint pacing (purple) and single‐point pacing (orange) during phase 2 of OPT‐MPP. Paired Student's *t*‐test. *, *p* = 0.015; ns, *p* > 0.05. [Colour figure can be viewed at wileyonlinelibrary.com]

At six months, LV contractility was similar at both resting and higher HRs during either programming modes (Table S3, Figure 4a). Whilst there was a trend to a higher critical HR at which peak contractility occurred with MPP, (MPP: mean 111 bpm [CI 99, 122] vs. SPP: 102 bpm [90, 115]; *p* = 0.28; Figure 4b), peak contractility itself trended to be lower with MPP programming (MPP: mean CCI 3.33 mmHg/mL/m^2^ [CI 2.12, 4.53] vs. SPP: 3.66 [2.22, 5.09]; *p* = 0.06; Figure 4c).

#### 3.2.2 Cardiopulmonary Exercise Testing

**FIGURE 5:**
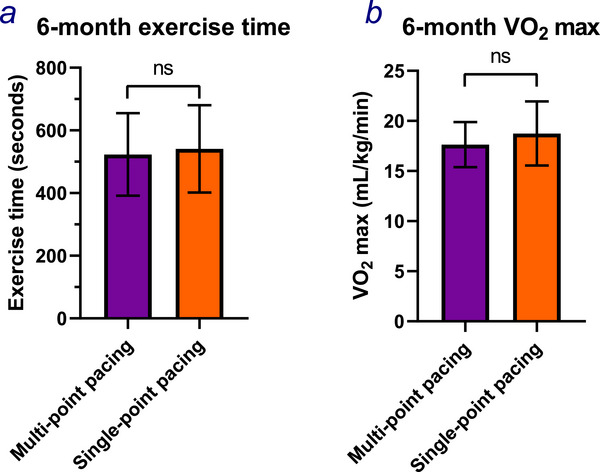
The differences in (a) the exercise time in seconds; (b) the maximal oxygen consumption between multipoint pacing (purple) and single‐point pacing (orange) during phase 2 of OPT‐MPP. Paired Student's *t*‐test. ns, *p* > 0.05. [Colour figure can be viewed at wileyonlinelibrary.com]

At six months, 17 of the 23 participants completed cardiopulmonary exercise testing. There were no differences in resting or peak HR, resting systolic or diastolic blood pressure, exercise time (Figure 5a), V̇O_2_ max (Figure 5b), OUES, anaerobic threshold, V̇E/VCO_2_ slope or V̇E/VCO_2_ slope to anaerobic threshold, or peak RER between tests performed in MPP or SPP modes (Table 3).

**TABLE 3:** The differences in cardiopulmonary exercise testing between multipoint and single‐point pacing during phase 2 of OPT‐MPP.

CPET, mean (95CI)	MPP	SPP	*p*‐value
Resting HR, bpm	68.8 (64.6, 73.1)	66.5 (59.6, 73.4)	0.54
Peak HR, bpm	105.7 (95.5, 115.9)	103.2 (93.1, 113.3)	0.71
Resting systolic BP, mmHg	125.0 (110.9, 139.1)	118.3 (104.8, 131.9)	0.40
Resting diastolic BP, mmHg	72.5 (61.1, 83.9)	66.7 (58.8, 74.6)	0.31
Exercise time, seconds	523.2 (391.6, 654.8)	541.0 (401.8, 680.2)	0.84
VO_2_ max, mL/kg/min	17.6 (15.4, 19.9)	18.7 (15.5, 21.9)	0.55
OUES	1717.4 (1335.0, 2099.7)	1830.1 (1500.4, 2159.9)	0.63
Anaerobic threshold	14.3 (12.2, 16.5)	14.8 (12.5, 17.1)	0.74
Ve/VCO_2_ slope	33.1 (27.7, 38.5)	33.8 (29.1, 38.4)	0.83
Ve/VCO_2_ slope to AT	28.2 (25.1, 31.3)	29.5 (25.6, 33.5)	0.57
Peak RER	1.13 (1.03, 1.22)	1.09 (1.03, 1.15)	0.47

*Note*: Paired Student's *t*‐test for statistical comparisons.

Abbreviations: CPET, cardiopulmonary exercise test; 95CI, 95% confidence interval; MPP, multipoint pacing; SPP, single‐point pacing; HR, heart rate; BP, blood pressure; OUES, oxygen uptake efficiency slope; AT, anaerobic threshold; RER, respiratory exchange ratio.

### 3.3 Phase 3

The baseline demographics of the 15 participants that completed the 6‐month parallel group study are described in Table 4. Overall, there was a reduction in exercise time in both groups over the 6 months. There was no difference in the change in exercise time between those allocated MPP and those randomized to SPP (MPP: ‐−81.9 s [CI −159.5, −4.2] vs. SPP: −41.5 [−104.7, 21.6]; *p* = 0.40, *F* = 0.76; Table 5) or V̇O_2_ max (MPP: 1.12 mL/kg/min [CI −2.71, 4.95] vs. SPP: −1.78 [−5.08, 1.53]; *p* = 0.24, *F* = 1.58). There were no differences in resting or peak HR, OUES, anaerobic threshold, V̇E/VCO_2_ slope or V̇E/VCO_2_ slope to anaerobic threshold, or peak RER after 6 months between those allocated MPP compared with those allocated SPP (Table 5).

**TABLE 4:** The baseline clinical and echocardiographic characteristics of the participants who were randomized to either multipoint pacing or single‐point pacing during phase 3 of OPT‐MPP.

Variable	Multipoint pacing (*n* = 6)	Single‐point pacing (*n* = 9)
Age (mean, 95CI), years	77.2 (72.6, 81.7)	72.0 (63.8, 80.2)
Male sex (*n*, %)	6 (100.0)	9 (100.0)
Height (mean, 95CI), m	1.73 (1.66, 1.79)	1.76 (1.70, 1.83)
Weight (mean, 95CI), kg	79.8 (73.6, 86.0)	84.4 (72.1, 96.7)
BSA (mean, 95CI), m^2^/kg	1.96 (1.85, 2.06)	2.03 (1.85, 2.20)
NYHA class at baseline (*n*, %)		
Class I	0	1 (11.1)
Class II	6 (100.0)	8 (88.9)
Class III	0	0
Class IV	0	0
Past medical history (*n*, %)		
Ischaemic heart disease	4 (66.7)	5 (55.6)
Peristent atrial fibrillation	3 (50.0)	3 (33.3)
Hypertension	3 (50.0)	3 (33.3)
Diabetes mellitus	1 (16.7)	4 (44.4)
Device type (*n*, %)		
CRT‐D	3 (50.0)	6 (66.7)
CRT‐P	3 (50.0)	3 (33.3)
Medical treatment (*n*, %)		
Beta‐blockers	6 (100.0)	9 (100.0)
ACE inhibitors/ARBs	6 (100.0)	9 (100.0)
MRA	2 (33.3)	5 (55.6)
Diuretics	5 (83.3)	6 (66.7)
Echocardiographic findings (mean, 95CI)		
LVEF, %	30.2 (20.5, 39.8)	31.2 (24.9, 37.5)
LVEDV, mL	166.3 (104.2, 228.3)	212.4 (153.5, 271.2)
LVESV, mL	120.3 (66.3, 174.4)	149.6 (100.4, 198.7)

Abbreviations: MPP, multipoint pacing; SPP, single‐point pacing; 95CI, 95% confidence interval; BSA, body‐surface area; NYHA, New York Heart Association; CRT‐D, cardiac resynchronization therapy defibrillator; CRT‐P, cardiac resynchronization therapy pacemaker; ACE, angiotensin‐converting enzyme; ARB, angiotensin II receptor blocker; MRA, mineralocorticoid receptor antagonist; LVEF, left ventricular ejection fraction; LVEDV, left ventricular end diastolic volume; LVESV, left ventricular end systolic volume.

**TABLE 5:** The differences in cardiopulmonary exercise testing between multipoint and single‐point pacing between phase 2 and phase 3 of OPT‐MPP.

CPET, mean (95CI)	MPP (*n* = 6)	SPP (*n* = 9)	*p*‐value
Resting HR, bpm	67.33 (53.77, 80.90)	71.56 (63.55, 79.56)	
Change in resting HR	−0.47 (−9.69, 8.76)	6.09 (−1.42, 13.59)	0.26
Peak HR, bpm	95.33 (79.59, 111.08)	106.56 (90.24, 122.87)	
Change in peak HR	−9.84 (−25.76, 6.07)	3.90 (−9.05, 16.84)	0.17

Exercise time, seconds	372.5 (203.6, 541.4)	571.9 (388.7, 755.0)	
Change in exercise time	−81.9 (−159.5, −4.2)	−41.5 (−104.7, 21.6)	0.40
VO_2_ max, mL/kg/min	16.70 (9.68, 23.72)	19.93 (15.52, 24.34)	
Change in VO_2_ max	1.12 (−2.71, 4.95)	−1.78 (−5.08, 1.53)	0.24

OUES	1548.50 (829.81, 2267.19)	1936.57 (1328.48, 2544.66)	
Change in OUES	80.44 (−151.33, 312.20)	−78.52 (−292.45, 135.41)	0.30
AT	13.35 (7.96, 18.74)	16.57 (10.65, 22.49)	
Change in AT	0.75 (−2.42, 3.92)	0.41 (−2.51, 3.34)	0.87

Ve/VCO_2_ slope	36.62 (22.42, 50.81)	30.97 (25.03, 36.91)	
Change in Ve/VCO_2_ slope	1.75 (−5.10, 8.61)	−2.53 (−8.86, 3.79)	0.34
Ve/VCO_2_ slope to AT	32.48 (24.42, 40.54)	28.43 (22.01, 34.85)	
Change in Ve/VCO_2_ slope to AT	4.61 (−2.70, 11.93)	−1.61 (−8.36, 5.14)	0.20
Peak RER	0.99 (0.86, 1.12)	1.00 (0.89, 1.11)	
Change in peak RER	−0.18 (−0.34, −0.02)	−0.11 (−0.26, 0.04)	0.52

*Note*: ANCOVA test for statistical comparisons in the change of values after 6 months adjusted for left ventricular ejection fraction, end diastolic volume, and end systolic volume.

Abbreviations: 95CI, 95% confidence interval; AT, anaerobic threshold; BP, blood pressure; CPET, cardiopulmonary exercise test; HR, heart rate; MPP, multipoint pacing; OUES, oxygen uptake efficiency slope; RER, respiratory exchange ratio; SPP, single‐point pacing.

## 4 Discussion

We found that activation of MPP soon after CRT implantation resulted in acutely improved LV contractility at resting HRs. However, this did not translate into improvements in the FFR or exercise capacity at 6‐weeks or 6‐months. Finally, 6 months of MPP was not associated with differences in measures of exercise capacity compared with SPP in our study.

### 4.1 The Force‐Frequency Relationship

The FFR describes the positive coupling of HR and LV contractility until a slope peak, beyond which contractility declines. In heart failure, this relationship is attenuated, such that contractility is reduced, as is the HR at which the slope peak occurs [15]. In patients with conventional CRT (SPP), we have previously demonstrated that optimizing device HR programming to take into account the individual's FFR translates into improvements in patient‐orientated outcomes such as walk distance, as well as preserving LV function [15]. Despite prior evidence showing significant echocardiographic improvements with MPP compared to conventional CRT without additional rate‐response programming [21, 22, 23], there are no data demonstrating improvements across the HR spectrum. Hence, OPT‐MPP contributes to our understanding of how this programming modality may or may not be advantageous.

### 4.2 Multi‐Point Pacing as a Programming Option

More than a third of patients who receive CRT with conventional SPP might be classified as nonresponders [24]. One strategy of improving response by potentially recruiting more of the lateral LV wall is to pace two cathodes simultaneously using quadripolar leads, known as MPP. This is hypothesized to improve LV contractility and thereby cardiac output by overcoming challenges such as variations in coronary venous anatomy, lead instability, or myocardial scar.

Recent trials have employed this strategy on CRT recipients who were nonresponders 6 months after standard biventricular SPP [25, 26] and looked at response rates and outcomes such as mortality and heart failure hospitalizations. Other prospective and registry‐based analyses have studied early MPP activation at the time of implantation [27, 28, 29] and have also focused on similar outcome measures. However, there is a lack of randomized data looking at the immediate effects of MPP on functional, patient‐oriented outcomes.

In our mechanistic crossover study, we observed improvements in contractility with activation of MPP shortly after implantation of the CRT devices. Although this was only present at resting HRs at the initial 6‐week assessment, it is consistent with invasive [8] and noninvasive [10] data showing an improvement in cardiac contractility at baseline HRs with early activation of MPP.

Beyond the acute response at resting HRs, the longer‐term data presented here support previous evidence that MPP is comparable to traditional SPP [30]. Compared to the data from 6 weeks, the CCI was higher in both the MPP and SPP groups at 6 months, which is consistent with what has been previously found [15]. There was no additional benefit of MPP in terms of LV contractility and exercise performance, both immediately following CRT, or over the longer term similar to previous data using LV end‐systolic volume and improvement in NYHA class as surrogates for LV contractility and exercise performance, respectively [11, 26]. Even attempts at ensuring targeted lead position to achieve maximal separation between LV pacing sites has demonstrated little additional benefit [26].

Finally, our data align with previous work showing that longer term hemodynamics are generally not further improved by MPP although there are suggestions that MPP might be associated with lower all‐cause mortality at 3 years [27], and in carefully selected CRT nonresponders, MPP might be associated with fewer heart failure hospitalizations after 6 months of MPP [31]. These studies enrolled patients with worse functional status at baseline than those in the present study. It is possible, therefore, that patients with more severe symptoms or those that are deteriorating might gain benefit from MPP.

### 4.3 The Longevity Dilemma

Finally, activation of MPP comes at a cost. We did not assess generator longevity and the need for generator replacement in the present study, yet previous work has demonstrated earlier generator depletion with a loss of between 1.1 and 1.9 years in CRT devices programmed to deliver MPP [13]. Given the risks of generator replacement, at present, there are no convincing data that MPP should routinely be activated and that optimized CRT with SPP should remain the programming of choice. Further work is required to decide whether the disparate data around MPP can be consolidated and thereby warrant a change to routine practice.

### 4.4 Limitations

This was a mechanistic study and as such no formal power calculation was performed. Therefore, we cannot discount the possibility of a type II error, although this risk is somewhat mitigated by the crossover study design. The Coronavirus disease 19 pandemic coincided with the follow‐up period of this study and, in addition to slow recruitment and a higher drop‐out rate than expected, also introduced variable durations of exposure to CRT due to difficulty in scheduling follow‐ups. The participant drop‐out at various stages of the study precluded paired statistical analyses. Most of our cohort consisted of stable patients with NYHA class II symptoms heart failure and it is possible that any benefits of MPP might be greater for patients with a worse or deteriorating clinical status. Further, there was no immediate post implantation echocardiogram done with the first imaging study performed at the point of randomization. Similarly, post implantation electrocardiographic data was not collected as our study was designed to assess the effects on patient‐oriented measures and on cardiac contractility. Finally, our assessment of the FFR was based on noninvasive methods for the measurement of contractility and although these have been previously well‐validated against invasive methods [32, 33], the results should be interpreted accordingly.

### 4.5 Conclusion

In unselected patients receiving CRT, activation of MPP did not translate into improvements in cardiac contractility or exercise performance. Based on these results, activating MPP during the initial phase of implantation in otherwise stable patients is not recommended as its effect is limited and may accelerate battery depletion. On the other hand, MPP might be of benefit in people who remain severely symptomatic or deteriorate despite CRT.

## Author Contributions

NZS, RMG, CAC, JEL, MFP and JG undertook the study procedures and collected the data. NZS and RMG analyzed the data. NZS and RMG produced the first draft of the manuscript. All other authors provided critical revision.

## Funding

This study was funded by an unconditional research grant from Medtronic.

## Conflicts of Interest

SS has received personal fees from Astra Zeneca. KKW has received personal fees from Medtronic, Cardiac Dimensions, Novartis, Abbott, BMS, Pfizer, Bayer and has received an unconditional research grant from Medtronic. MTK has received personal fees and an unselected research grant from Astra Zeneca, and an unrestricted research grant from Medtronic. JG has received personal fees from Abbott, Medtronic and Microport and has received an unrestricted research grant from Medtronic. None of the other authors have conflicts of interest to declare.

## Supporting information




**Supplementary Table 1**: Cardiac resynchronization therapy device information.**Supplementary Table 2**: The differences in the force frequency relationship between multipoint and single‐point pacing during phase 1 of OPT‐MPP. **Supplementary Table 3**: The differences in the force frequency relationship between multipoint and single‐point pacing during phase 2 of OPT‐MPP.

## Data Availability

The datasets generated from this study are not publicly available due to the inclusion of potentially identifiable information but are available in anonymized form from the corresponding author upon reasonable request.
