# Supplementary material for: Exercise Capacity and the Force Frequency Relationship in Multi‐Point Versus Single‐Point Pacing: A Randomized Trial
Source: Pacing Clin Electrophysiol. 2026 Feb 20;49(6):685–97. doi: 10.1111/pace.70164 (PMC13238297; doi:10.1111/pace.70164)
Supplement: Supplementary file 1 — Supplementary Table 1: Cardiac resynchronization therapy device information.Supplementary Table 2: The differences in the force frequency relationship between multipoint and single‐point pacing during phase 1 of OPT‐MPP. Supplementary Table 3: The differences in the force frequency relationship between multipoint and single‐point pacing during phase 2 of OPT‐MPP. [file PACE-49-685-s001.docx]

**SUPPLEMENTARY MATERIALS**

Supplementary Table 1: Cardiac resynchronisation therapy device information.

| **Patient** | **Generator model (manufacturer)** | **Left ventricular lead model (manufacturer)** |
| --- | --- | --- |
| Patient 1 | Amplia DTMB2QQ (Medtronic) | Attain Stability Quad 479888 (Medtronic) |
| Patient 2 | Amplia DTMB2QQ (Medtronic) | Attain Stability Quad 479888 (Medtronic) |
| Patient 3 | Amplia DTMB2QQ (Medtronic) | Attain Performa 4298 (Medtronic) |
| Patient 4 | Percepta Quad W4TR04 (Medtronic) | Attain Performa 4298 (Medtronic) |
| Patient 5 | Percepta Quad W4TR04 (Medtronic) | Attain Performa 4298-78 (Medtronic) |
| Patient 6 | Amplia DTMB2QQ (Medtronic) | Attain Performa 4298 (Medtronic) |
| Patient 7 | Quadra Assura MP CD3371-40QC (Abbott) | Quartet 1458QL-86 (Abbott) |
| Patient 8 | Quadra Allure MP PM3562 (Abbott) | Quartet 1458Q-86 (Abbott) |
| Patient 9 | Percepta Quad W4TR04 (Medtronic) | Capsure Sense 4574 45 (Medtronic) |
| Patient 10 | Percepta Quad W4TR04 (Medtronic) | Attain Performa 4298-78 (Medtronic) |
| Patient 11 | Amplia DTMB2QQ (Medtronic) | Attain Performa 4298-78 (Medtronic) |
| Patient 12 | Quadra Allure MP PM3562 (Abbott) | Quartet 1456Q 86 (Abbott) |
| Patient 13 | Quadra Allure MP PM3562 (Abbott) | Quartet 1456Q 86 (Abbott) |
| Patient 14 | Amplia DTMB2QQ (Medtronic) | Attain Performa 4298-78 (Medtronic) |
| Patient 15 | Percepta Quad W4TR04 (Medtronic) | Attain Stability Quad 4798 78 (Medtronic) |
| Patient 16 | Compia MRI CRTD DTMC2QQ (Medtronic) | Attain Performa 4298-78 (Medtronic) |
| Patient 17 | Amplia DTMB2QQ (Medtronic) | Attain Stability Quad 4798 88 (Medtronic) |
| Patient 18 | Amplia DTMB2QQ (Medtronic) | Attain Stability Quad 4798 78 (Medtronic) |
| Patient 19 | Amplia DTMB2QQ (Medtronic) | Attain Stability Quad 4798 78 (Medtronic) |
| Patient 20 | Quadra Assura MP CD3371-40QC (Abbott) | Quartet 1456Q 86 (Abbott) |
| Patient 21 | Amplia DTMB2QQ (Medtronic) | Attain Stability Quad 4798-78 (Medtronic) |
| Patient 22 | Quadra Allure MP PM3562 (Abbott) | Tendril STS 2088TC-46 (Abbott) |
| Patient 23 | Amplia DTMB2QQ (Medtronic) | Attain Stability Quad 4798 88 (Medtronic) |

Supplementary Table 2: The differences in the force frequency relationship between multi-point and single-point pacing during phase 1 of OPT-MPP.

| **Heart rate (bpm), mean (95CI)** | **Cardiac contractility index (mmHg/ml/m^2^) with MPP** | **Cardiac contractility index (mmHg/ml/m^2^) with SPP** | **p-value** |
| --- | --- | --- | --- |
| 65 | 2.29 (1.74, 2.84) | 2.03 (1.58, 2.47) | 0.019 |
| 80 | 2.42 (1.82, 3.02) | 2.23 (1.78, 2.69) | 0.076 |
| 95 | 2.51 (1.89, 3.14) | 2.54 (1.88, 3.20) | 0.88 |
| 110 | 2.47 (1.86, 3.08) | 2.45 (1.87, 3.02) | 0.82 |
| 125 | 2.43 (1.86, 3.01) | 2.37 (1.81, 2.92) | 0.30 |
| 140 | 2.46 (1.84, 3.08) | 2.42 (1.78, 3.06) | 0.67 |
| bpm, beats per minute; 95CI, 95% confidence interval; MPP, multi-point pacing; SPP, single-point pacing.  Paired Student’s T-test for statistical analysis. | | | |

Supplementary Table 3: The differences in the force frequency relationship between multi-point and single-point pacing during phase 2 of OPT-MPP.

| **Heart rate (bpm), mean (95CI)** | **Cardiac contractility index (mmHg/ml/m^2^) with MPP** | **Cardiac contractility index (mmHg/ml/m^2^) with SPP** | **p-value** |
| --- | --- | --- | --- |
| 65 | 2.44 (1.29) | 2.54 (1.33) | 0.54 |
| 80 | 2.68 (1.34) | 2.79 (1.65) | 0.42 |
| 95 | 2.70 (1.38) | 2.83 (1.48) | 0.36 |
| 110 | 2.78 (1.36) | 3.02 (1.91) | 0.26 |
| 125 | 2.98 (2.51) | 3.37 (2.92) | 0.015 |
| 140 | 3.17 (2.13) | 3.43 (2.88) | 0.37 |
| bpm, beats per minute; 95CI, 95% confidence interval; MPP, multi-point pacing; SPP, single-point pacing.  Paired Student’s T-test for statistical analysis. | | | |
